# Odor Perception in Children with Autism Spectrum Disorder and its Relationship to Food Neophobia

**DOI:** 10.3389/fpsyg.2015.01830

**Published:** 2015-12-01

**Authors:** Anne-Claude Luisier, Genevieve Petitpierre, Camille Ferdenzi, Annick Clerc Bérod, Agnes Giboreau, Catherine Rouby, Moustafa Bensafi

**Affiliations:** ^1^Research Center in Neurosciences of Lyon, CNRS UMR5292, INSERM U1028, Claude Bernard University Lyon 1Lyon, France; ^2^Senso5 FoundationSion, Switzerland; ^3^Institute of Special Education, University of FribourgFribourg, Switzerland; ^4^Center for Food and Hospitality Research, Institut Paul BocuseEcully, France

**Keywords:** autism, olfaction, food neophobia, hedonic evaluation, exploratory behavior

## Abstract

Atypical sensory functioning in Autism Spectrum Disorder (ASD) has been well documented in the last decade for the visual, tactile and auditory systems, but olfaction in ASD is still understudied. The aim of the present study was to examine whether children with ASD and neuro-typically (NT) developed children differed in odor perception, at the cognitive (familiarity and identification ability), sensorimotor (olfactory exploration) and affective levels (hedonic evaluation). Because an important function of the sense of smell is its involvement in eating, from food selection to appreciation and recognition, a potential link between odor perception and food neophobia was also investigated. To these ends, 10 children between 6 and 13 years old diagnosed with ASD and 10 NT control children were tested. To compare performance, 16 stimuli were used and food neophobia was assessed by the parents on a short food neophobia scale. Results revealed that (i) significant hedonic discrimination between attractive and aversive odors was observed in NT (*p* = 0.005; *d* = *2.378*) and ASD children (*p* = 0.042; *d* = 0.941), and (ii) hedonic discrimination level was negatively correlated with food neophobia scores in ASD (*p* = 0.007) but not NT children. In conclusion, this study offers new insights into odor perception in ASD children, highlighting a relationship between odor hedonic reactivity and eating behavior. This opens up new perspectives on both (i) the role of olfaction in the construction of eating behavior in ASD children, and (ii) the measurement and meaning of food neophobia in this population.

## Introduction

According to the American Psychiatric Association’s Diagnostic and Statistical Manual, Fifth Edition (DSM-5), Autism Spectrum Disorder (ASD) is characterized by both (i) deficits in social communication and social interaction and (ii) stereotyped, restricted, repetitive patterns of behavior, interest or activity (including atypical speech and movement, resistance to change, and atypical sensory behavior). These symptoms are present in early childhood and combine to limit and impair everyday functioning.

Atypical sensory functioning in ASD has been well documented in the last decade for the visual (Simmons et al., 2009), tactile (Puts et al., 2014) and auditory (Hitoglou et al., 2010; O’Connor, 2012) systems (Marco et al., 2011); for instance, it has been shown that orientation toward social sounds is impaired in ASD children (Dawson et al., 2004). On the other hand, olfaction and taste in ASD are still understudied despite the fact that experimental proof of the importance of environmental odor cues for the social and cognitive development of ASD children was provided by two recent studies. In the first, Parma et al. (2013) showed that automatic imitation – a prominent social skill that is impaired in ASD – in a reach-to-grasp action task is induced in ASD children when the object to be grasped is paired with the smell of their own mother, suggesting that a familiar body odor may promote imitation in ASD children. In the second study, Woo and Leon (2013) exposed 3–12 year-old ASD children to either daily olfactory/tactile stimulation along with sensory and cognitive exercises (enrichment group), or to only standard care (control group); after 6 months of enrichment, the severity of autistic traits (assessed on the Childhood Autism Rating Scale, Schopler et al., 1980) was significantly lower in the enrichment group than in controls.

Besides these 2 promising scientific attempts, the few clinical and scientific reports available that characterized olfactory function in this population suggest that individuals with ASD have atypical responses to olfactory stimuli (reviewed in Schecklmann et al., 2013 and Martin and Daniel, 2014), although results have not often been concordant: odor detection ability was equivalent between adults with ASD and controls in three studies (Suzuki et al., 2003; Tavassoli and Baron-Cohen, 2012; Galle et al., 2013), whereas in another study odor detection was better in ASD patients (Ashwin et al., 2014). Odor identification was impaired in two studies (Suzuki et al., 2003; Galle et al., 2013). Studies in ASD children also showed lack of consensus: Bennetto et al. (2007) reported lower odor identification ability in ASD patients than controls, whereas Dudova et al. (2011) found lower odor detection but no difference in identification between ASD children and healthy controls.

However, olfaction has important functions involving other abilities than just detection and identification, and these functions have been understudied in ASD patients. Firstly, the sense of smell constitutes an early warning system against odorant molecules that may, for example, signal toxic food to be avoided. Secondly, it plays a major role in hedonic pleasure, especially regarding food. Hrdlicka et al. (2011) showed that ASD children perceived the odors of pineapple and cinnamon (among 16 odors) as less pleasant than controls; but how hedonic ratings is changed for pleasant odors and unpleasant odors in ASD children remains unclear. Are these important functions of olfaction (attraction to and avoidance of smells) enhanced/maintained/impaired in this disorder? The general aim of the present study was to characterize olfactory function in ASD children at both the cognitive (odor familiarity and odor identification ability: **objective 1**) and sensorimotor and hedonic levels (**objective 2**), by considering the positive and negative hedonic value of smells. To this end, a pleasant and an unpleasant odor (at various concentrations) selected from a previous study (Joussain et al., 2013) were presented to ASD children and controls. The odors were embedded in a series of 16 stimuli including a non-odorized stimulus and odorant compounds that included both mixtures of molecules and their individual components. Whereas no hypothesis was tested for the mixtures and the individual components, for pleasant and unpleasant stimuli, we tested the bidirectional hypothesis that affective reactivity to odors is reflected by (i) hypo-emotionality (decreased pleasantness of attractive odors; decreased unpleasantness of aversive odors) or (ii) hyper-emotionality (increased pleasantness of attractive odors; increased unpleasantness of aversive odors). Verbal responses were collected, accompanied by behavioral quantification of nasal olfactory exploration using video tools.

An important function of the sense of smell is its involvement in eating, from food selection to appreciation and recognition. Eating is a multifactorial mechanism involving three main sources of variability: the eater (with his/her food history and sensations), the object (food and its characteristics) and the context (physical and social environment Rozin and Tuorila, 1993; Meiselman and MacFie, 1996; Renner et al., 2012). Eating activities have become more complex over the course of evolution and the determinants of food choice are multiple (Köster, 2009). Eating well (or normally) can be learned. The construction of children’s dietary behavior requires sensorimotor, social and psychological skills (de Suremain and Razy, 2012). The process is sometimes difficult: eating disorders affect 13–50% of neuro-typically (NT) developed children, but more than 80% of children with ASD (Ledford and Gast, 2006; Nadon et al., 2013). In particular, selectivity is by far the most common problem encountered by children with ASD (Sharp et al., 2013; Cermak et al., 2014; Rastam and Wentz, 2014).

Although the term “food selectivity” has been understood in different ways in *ad hoc* studies of ASD children, there is some consensus that it restricts the number of accepted foods (Cermak et al., 2014; Rastam and Wentz, 2014). The primary objective of food learning is to widen the diversity of foods accepted by children, so as at least to cover their vital needs. This opening strengthens and widens during childhood and adolescence (Nicklaus, 2009). Many intrinsic and extrinsic factors influence the acceptance of new foods by children, such as parental behavior or sensory processes (Blissett and Fogel, 2013). A major hindrance to widening food diversity and the acceptance of new foods is **food neophobia**, defined as a reluctance to consume or tendency to reject foods considered new by the eater (Loewen and Pliner, 1999; Dovey et al., 2008). Food neophobia was found to be associated with sensory experience (Aldridge et al., 2009; Shim et al., 2011), sensory functioning (Cooke, 2007) and anxiety (Galloway et al., 2003).

One of the main causes of greater food selectivity in children with ASD may lie in their particular sensory functioning (Matson and Fodstad, 2009; Beighley et al., 2013; Cermak et al., 2014). Notably, olfactory alterations may jeopardize acceptance of food and dangerously restrict variety of diet in ASD children (Demattè et al., 2014). Therefore, the third objective (**objective 3**) of the present study was to examine the relationship between hedonic response to pleasant and unpleasant odors and behavioral attitudes toward food (i.e., food neophobia).

## Materials and Methods

### Participants

This preliminary study, approved by the *Commission Cantonale Valaisanne d’Ethique Médicale* institutional review board (IRB number: CCVEM 022/14), tested 10 children diagnosed with ASD (all boys; age range, 6–13 years) and 10 NT control children, matched for age (±6 months) and gender. The ASD group was composed with children considered as eligible for the Swiss ASD Observatory and children officially diagnosed by the Autism Diagnostic Assessment Centre of Lyon. No data were available on IQ and language level. With regard to ASD symptom, six were announced with ASD or with pervasive developmental disorder and four as Asperger. The NT control participants had normal school performance, without any known behavioral or psychological disorder. All participants and their legal guardians agreed to participate in the study by signing a consent form.

Food neophobia was assessed by the parents on a standard 10-item questionnaire (the French adapted food neophobia scale: AFNS) with good internal consistency (Reverdy et al., 2008). For each item, parents were required to indicate to what extent the corresponding statement was true, on a 7-point scale from “Very true for me” to “Not at all true for me.”

The 10 items were: (1) My son is very particular about the foods he will eat (reversed scoring); (2) My son likes foods from different countries; (3) My son doesn’t trust new foods (reversed scoring); (4) My son likes to try unusual foods; (5) When my son has the choice between different flavors for a certain food (for example, ice-cream or sweets), he likes to choose a flavor that he doesn’t not know; (6) My son will try a dish, even if he doesn’t not know what’s in it; (7) The foods my son knows are sufficient for him (reversed scoring); (8) My son is willing to eat anything that is offered; (9) My son is afraid to eat things he has never had before (reversed scoring); and (10) My son will not taste a food when he doesn’t know what it is (reversed scoring). For questions 2, 4, 5, 6, and 8, the highest score (7 points) was given to the response “Very true for my son” and the lowest (1 point) to “Not at all true for my son”; for questions 1, 3, 7, 9, and 10, the scores were reversed. The food neophobia score was obtained by adding the scores for the 10 questions (range: 10–70); the higher the score, the higher the neophobia grade.

There was no significant difference between groups in terms of age in years (mean ± SEM; NT: 9.97 ± 0.80, ASD: 9.58 ± 0.83; Mann-Whitney test: *z* = 0.680, *p* > 0.05) or food neophobia score (NT: 48.8 ± 4.27, ASD: 42.4 ± 4.75; Mann–Whitney test: *z* = 0.869, *p* > 0.05).

### Stimuli

In order to compare hedonic reactivity to pleasant and unpleasant food odors in ASD and NT children, 4 concentrations of a pleasant mint odor (L-Carvone, CID = 439570, 1%, 2.37%, 5% and 10%) and unpleasant fishy odor (Trimethylamine, CID = 1146, 10, 25, 50, and 100%) were presented to the participants. In addition, three binary mixtures (50/50%) containing respectively the smells of (rose + grass), (vanilla + cocoa) and (rose + cocoa), and their individual components (“vanilla”: ethyl vanillin, CID = 8467, 100%; “cocoa”: isobutyl phenylacetate, CID = 60998, 28%; “rose”: phenyl ethanol, CID = 6054, 2.65%; and “grass”: cis-3-hexenol, CID = 5281167, 0.21%) were also presented. All odorants (Sigma–Aldrich) were diluted in mineral oil. They were presented in 15 ml flasks (opening diameter: 1. 7cm; height: 5.8 cm; filled with 5 ml dilution) and absorbed on scentless polypropylene fabric (3 cm × 7 cm; 3 M, Valley, NE, USA) to optimize evaporation and air/oil partitioning. Finally, an empty jar containing only an odorless solvent (mineral oil) served as control stimulus. A total of 16 stimuli (15 odorous and 1 control) were thus used.

### Protocol

One important aspect of children’s involvement in the study was that they were prepared for the experimental sessions a few weeks before. They had been informed in advance by their teacher and parents that they would take part in a sensory study involving olfaction. Experiments were performed in the cities of Sion and Sierre (Switzerland), in specially adapted rooms.

The experimenter started with a detailed explanation of the procedure to the child. Participants were required to sit on a chair, either on the right or left side of the experimenter, in front of a table (or if not possible, with a box on their knees). They were videotaped by two digital camcorders (one in front of the participant, and the other oriented toward his left or right profile) during the experimental session. The experiment started as soon as the participant was installed, and included two phases.

Phase 1 consisted in familiarizing the children with olfactory exploration. Sixteen trials were presented in randomized order (Hasard software). The experimenter opened a jar and gave it to the child, who was asked to smell the odor, without touching the odorant jar with his nose, and to put the jar back on the table or in the box once smelled. Stimulus-onset asynchrony varying from 20 to 30 s was used.

Phase 2, the experimental phase, was conducted the same day, at least 30 min after phase 1. Verbal and behavioral responses to the same 16 stimuli were characterized in ASD and NT children using implicit (video recording of olfactory exploration) and explicit (verbal response) approaches. As in phase 1, each trial started as soon as the experimenter presented the jar to the child, telling him: “You have to smell this jar without touching it with your nose.” The child’s task was to answer the following questions: (1) “Do you like this odor?”; (2) “Do you know what is it?”; and (3) “Can you tell me what it is?”. Stimulus-onset asynchrony from 20 to 90 s was used, depending on the child’s verbal production.

### Data Analysis

#### Verbal Data

The first question (“Do you like this odor?”) enabled analysis of hedonic response, scored as follows:

“1” for a “Yes” or nod of the head or any positive response such as “It’s ok,” “It’s good,” etc.; “-1” for a “No” or any negative response such as “Not so much,” “Not really,” “Not too much,” etc.; or “0” for an unclear or non-hedonic response such as “I don’t know,” “Medium,” “Strong,” “So-so,” “Quite strong,” “Strong, medium,” etc.

The second question (“Do you know what it is?”) enabled analysis of odor familiarity, scored as “1” for a “Yes,” and “0” otherwise.

Finally, the third question (“Can you tell me what it is?”) enabled analysis of identification ability, coded by conformity with a veridical label (vl). One or several vls were defined for each odor, with a score of “1” if any of the vls was used; however, if the participant did not use the vls, but used a semantically related word, then 0.5 point was affected: (1) L-Carvone (four concentrations; vl = “Mint,” but “Toothpaste” accepted); (2) Trimethylamine (four concentrations; vl = “Fish,” but “Pooh,” “Anchovy,” or “Cat-food” accepted); (3) Phenyl ethanol (vl = “Rose,” with 0.5 points for “Lavender” or “Herbs,” as being semantically close); (4) Cis-3 hexenol (vl = “Grass,” with 0.5 points for “Grape” or “Crushed flowers”); (5) Ethyl vanillin (vl = “Vanilla” and/or “Caramel,” with 0.5 points for “Sugar”); (6) Phenyl acetate isobutyl (vl = “Chocolate” or “Cocoa”); (7) Phenyl ethanol + Cis-3-hexenol (vl = “Rose,” “Flower” or “Grass,” with 0.5 points for “Grape,” “Leaf” or “Herbs”); (8) Ethyl vanillin + Phenyl acetate isobutyl (vl = “Vanilla,” “Caramel,” “Chocolate” or “Cocoa,” with 0.5 points for “Honey”); (9) Phenyl ethanol + Ethyl vanillin (vl = “Flower,” “Rose,” “Caramel” or “Vanilla”), (10) solvent (no vl).

#### Behavioral Data

The profile video sequence recorded for each participant was divided into 16 segments, corresponding to each odorant condition, using appropriate software (Volcan^®^, Lyon, France; see Rinck et al., 2011). For each segment, olfactory exploration of the jar was quantified, starting when the participant moved the jar in front of his nose/lip, or even earlier if a strong focus of the odor was observed (e.g., head movement or marked diminution of the approach movement), and ending when the participant moved the jar away from his nose. Four variables were analyzed: (i) number of olfactory explorations per stimulus; (ii) total duration of olfactory exploration; (iii) mean duration of olfactory exploration (total duration/number of explorations); and (iv) duration of the first olfactory exploration.

### Statistical Analyses

For statistical analyses of verbal and behavioral data, five parameters were calculated for each participant and each variable (verbal variables: odor identification, odor familiarity, and odor pleasantness; behavioral variables: number of olfactory explorations, total duration of exploration, mean duration of exploration, and duration of first exploration): (1) mean value for all 15 odors (m_g_); (2) mean value for the four trials of L-Carvone (m_L-Carvone_); (3) mean value for the four trials of Trimethylamine (m_Trimethylamine_); (4) mean value for all simple mixture components (m_simple_); and (5) mean value for all mixtures (m_mixture_). It is important to note here that 50% of the ASD children were not able (or did not agree) to perform the whole study (see Results), so that, because the experimental design was randomized, the mean value calculated for each odor category (carvone or trimethylamine, for example) was not necessarily based on the same number of trials; consequently, the effect of odor concentration for carvone and trimethylamine could not be assessed.

Two types of statistical comparison were performed: (1) inter-group comparison between NT ASD groups for the parameters m_g_, m_L-Carvone_, m_Trimethylamine_, m_simple_ and m_mixture_ used Mann–Whitney *U* tests for all verbal and behavioral variables; (2) intra-group comparison of m_L-Carvone_ vs. m_Trimethylamine_ and m_simple_ vs. m_mixture_, in the NT group on the one hand and in the ASD group on the other hand, used Wilcoxon tests.

Finally, to relate odor hedonic perception with food neophobia, two types of correlation analysis were performed: (i) between pleasantness ratings of both pleasant and unpleasant odors on the one hand, and food neophobia score on the other hand; and (ii) between a hedonic categorization index (the absolute value of the difference between the mean hedonic score for L-Carvone (m^h^_L-Cavone_) and the mean hedonic score for Trimethylamine (m^h^_Trimethylamine_) (i.e., m^h^_L-Cavone_ – m^h^_Trimethylamine_) on the one hand and food neophobia score on the other hand.

For all analyses, the level of statistical significance was set at 0.05. Analyses were performed using SPSS software (version 22 for Windows).

## Results

Firstly, as regards the experiment itself, NT children were able to perform the whole experimental session (16 odors), whereas ASD children were not able to experience all the odorant stimuli during the session (mean ± SEM; 12.8 ± 1.21; trend on Mann–Whitney test: *z* = 1.890, *p* = 0.058). The interruption was made at the child’s request, for the following reasons: one child decided from the outset to test only eight odors; one child could no longer concentrate; and three children expressed emotional reactions such as disgust, preventing them from continuing.

All statistics (*z* and *p* values) for identification, familiarity, pleasantness and behavioral data are presented in **Tables 1** and **2**.

**Table 1:** Inter-group and intra-group comparison for odor identification, familiarity and pleasantness: sample size (*N*), observed *z*-value and *p*-value for each parameter and each variable.

	Identification score			Familiarity ratings			Hedonic value		
	*N*	*z-value*	*p-value*	*N*	*z-value*	*p-value*	*N*	*z-value*	*p-value*
m_g_	18	-0.535	0.593	18	-0.936	0.349	18	-0.089	0.929
m_L-Carvone_	18	-1.331	0.183	18	-0.552	0.581	18	-1.115	0.265
m_Trimethylamine_	18	-0.320	0.749	18	-1.206	0.228	18	-0.239	0.811
m_simple_	18	-0.183	0.855	18	-0.819	0.413	18	-0.584	0.559
m_mixture_	17	-0.872	0.383	17	-0.253	0.800	17	-0.546	0.585
**m_**L-Carvone**_ vs. m_**Trimethylamine**_**								
NT	10	-1.697	**0.090**	10	-1.491	0.136	10	-2.816	**0.005**
ASD	8	-0.216	0.829	8	-0.184	0.854	8	-2.032	**0.042**
**m_**simple**_ vs. m_**mixture**_**								
NT	10	-0.831	0.406	10	-0.211	0.833	10	-1.472	0.141
ASD	7	-0.422	0.673	7	-1.355	0.176	7	-1.892	**0.058**

*p-values < 0.1 are highlighted.*

**Table 2:** Inter-group and intra-group comparison for number of olfactory explorations, total duration of exploration, mean duration of all explorations and duration of first exploration: sample size (*N*), observed *z*-value and *p*-value for each parameter and each variable.

		Number of olfactory explorations		Total duration of exploration		Mean duration of all explorations		Duration of first exploration	
	*N*	*z-value*	*p-value*	*z-value*	*p-value*	*z-value*	*p-value*	*z-value*	*p-value*
m_g_	19	-1.643	0.100	-0.653	0.514	-1.388	0.165	-1.306	0.191
m_L-Carvone_	19	-0.178	0.859	-0.163	0.870	-1.061	0.288	-1.143	0.253
m_Trimethylamine_	19	-1.464	0.143	-1.143	0.253	-1.715	**0.086**	-1.960	**0.050**
m_simple_	19	-1.152	0.249	-0.041	0.967	-0.408	0.683	-0.327	0.744
m_mixture_	18	-1.274	0.203	-0.889	0.374	-1.510	0.130	-0.933	0.351
**m_**L-Carvone**_ vs. m_**Trimethylamine**_**								
NT	10	0.0001	0.999	-0.153	0.878	-0.255	0.799	-0.255	0.799
ASD	9	-1.089	0.276	-0.533	0.594	-1.599	0.110	-1.836	**0.066**
**m_**simple**_ vs. m_**mixture**_**								
NT	10	-0.368	0.713	-0.153	0.878	-0.357	0.721	-0.357	0.721
ASD	8	-1.270	0.204	0.0001	0.999	-0.420	0.674	-0.420	0.674

*p-values < 0.1 are highlighted.*

**FIGURE 1:**
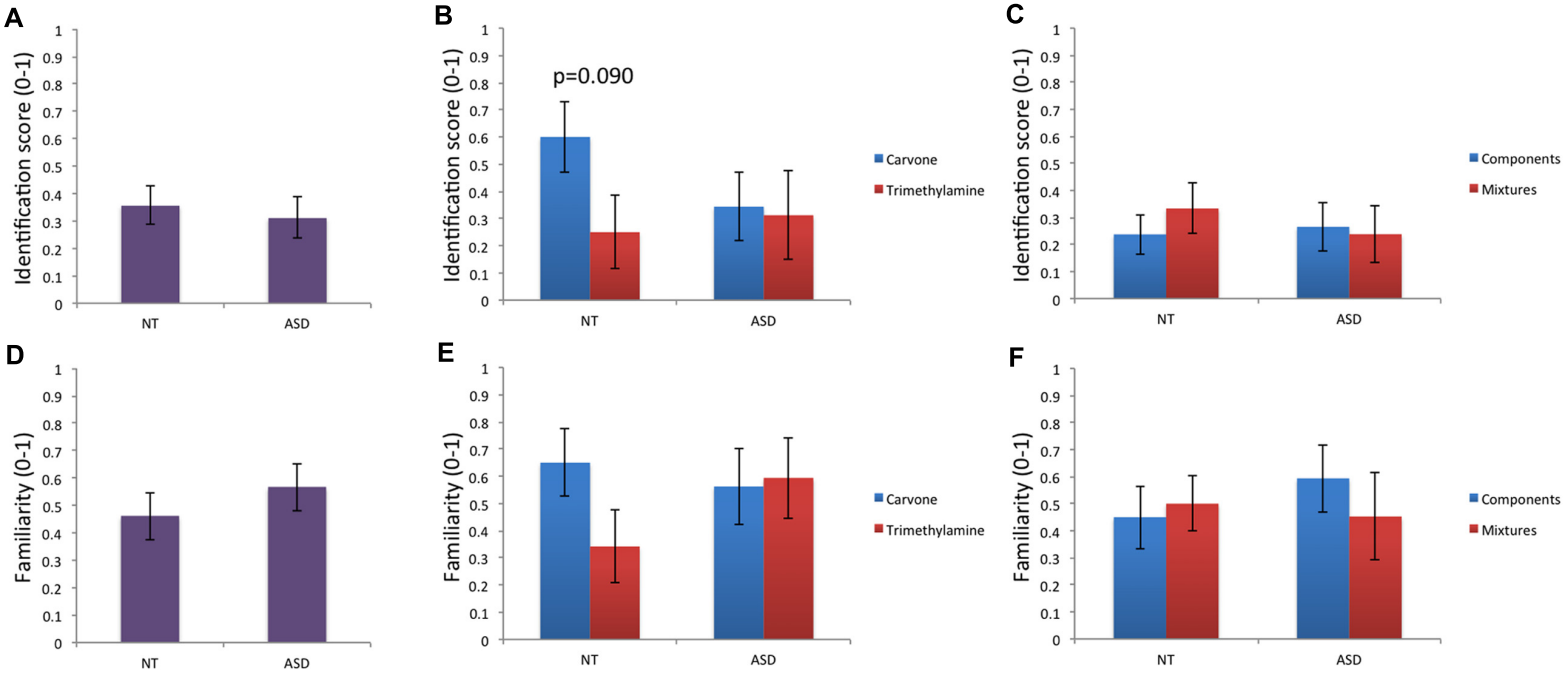
**Odor identification and odor familiarity. (A–C)** Odor identification performance did not differ between groups or between odor conditions within groups. A trend (*p* < 0.10) toward better identification of the pleasant odor (Carvone) than the unpleasant odor (Trimethylamine) was observed in the NT group. **(D–F)** For familiarity ratings, no significant effect of group or odor condition (within group) was observed.

Regarding verbal data, analysis of odor identification performance (**Table 1**; **Figures 1A–C**) revealed no significant effect of group for m_g_, m_L-Carvone_, m_Trimethylamine_, m_simple_, or m_mixture_. On intra-group comparison, a trend toward better identification of the pleasant odor Carvone than the unpleasant odor Trimethylamine was observed in the NT but not in the ASD group, while comparison between mixtures and their individual components was not significant in either NT or ASD children.

Regarding familiarity ratings (**Table 1**; **Figures 1D–F**), no significant difference between groups was found for m_g_, m_L-Carvone_, m_Trimethylamine_, m_simple_, or m_mixture_. Moreover, Carvone and Trimethylamine did not differ in familiarity in the NT or ASD group; nor did the mixtures and their individual components.

**FIGURE 2:**
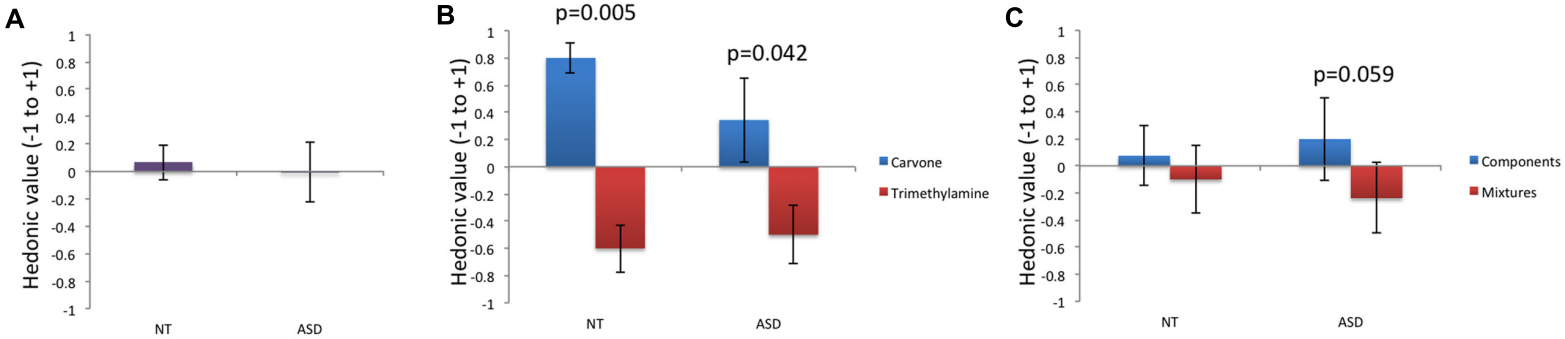
**Odor pleasantness.** For odor pleasantness ratings, no difference was observed between groups when considerign all odors **(A)**. A significant effect of valence was observed in the NT group: the smell of Carvone was perceived as significantly more pleasant than the smell of Trimethylamine (*p* = 0.005). A similar effect of valence was observed in the ASD group, although the magnitude of the effect was lower (*p* = 0.042) **(B)**. It noteworthy that a trend toward lower pleasantness of odor mixtures than the individual components was observed in the ASD group (*p* = 0.059) **(C)**.

With regard to odor pleasantness (**Table 1**; **Figure 2**), no significant effect of group was observed for m_g_, m_L-Carvone_, m_Trimethylamine_, m_simple_, or m_mixture_, but intra-group comparison revealed that Carvone was rated as significantly more pleasant than Trimethylamine by NT children, and by ASD children. To assess the magnitude of this effect in each group, we performed an effect size analysis using Cohen’s *d* for paired samples. Results obtained with a classical bootstrap procedure (1000 resamples for each group) showed that effect size was greater in NT (Cohen’s *d*: 2.378; Percentile Bootstrap 95% Confidence Interval or CI: 1.709–4.487) than in ASD (Cohen’s *d*: 0.941; Percentile Bootstrap 95% CI: 0.503–1.881), although the two CI overlapped slightly.

Moreover, whereas mixtures and their individual components did not differ in pleasantness in the NT group, there was a trend toward lower pleasantness for mixtures than the components in the ASD group.

**FIGURE 3:**
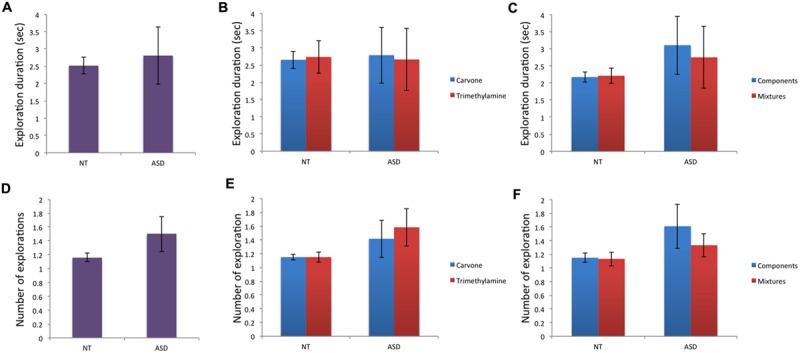
**Behavioral data (i): total duration of explorations and number of olfactory explorations. (A–C)** No significant effect of group or of odor conditions within groups was observed for total duration of exploration. **(D–F)** For number of olfactory explorations, no significant effect of group or odor condition (within group) was observed.

Regarding behavioral data (**Table 2**): for the variable “total duration of exploration” (**Figures 3A–C**), no significant effect of group was found for m_g_, m_L-Carvone_, m_Trimethylamine_, m_simple_, or m_mixture_ and intra-group comparison did not show any significant difference between Carvone and Trimethylamine in the NT or ASD group. Moreover, no significant difference between mixtures and their individual components was observed in the NT or ASD group.

Analysis of “number of olfactory explorations” (**Figures 3D–F**) found no significant effect of group for m_g_, m_L-Carvone_, m_Trimethylamine_, m_simple_, or m_mixture_. Intra-group comparison found no significant difference between Carvone and Trimethylamine, or between mixtures and their individual components, in the NT or ASD group.

**FIGURE 4:**
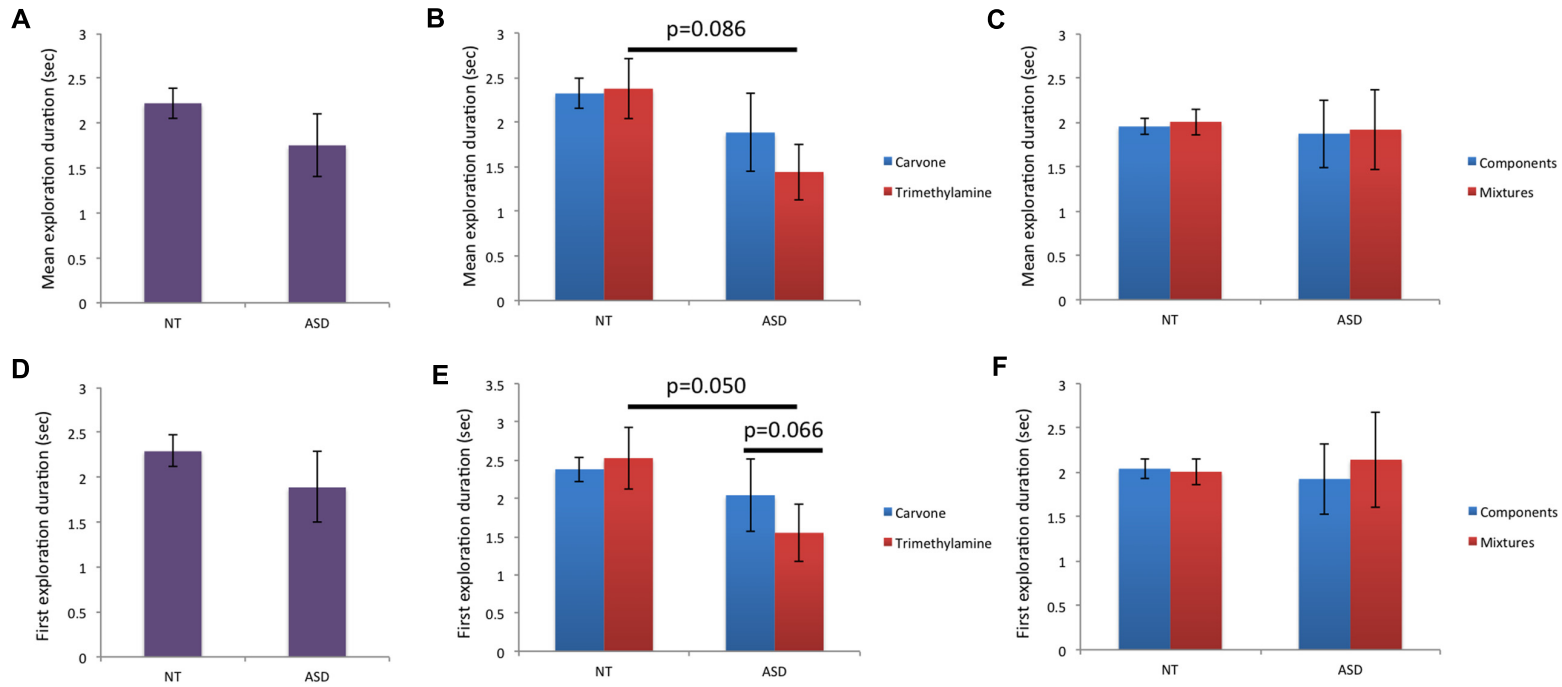
**Behavioral data (ii): mean duration of all explorations and duration of first exploration. (A–C)** For mean duration of all explorations, a trend toward shorter mean duration for the unpleasant smell (Trimethylamine) in ASD than NT children was found (*p* = 0.086). The other inter-group or intra-group comparisons were non-significant. **(D–F)** For duration of first exploration, a significant effect of group was observed for Trimethylamine: ASD children explored this unpleasant odor less than NT children (*p* = 0.050). Moreover, a trend toward shorter exploration duration for Trimethylamine than Carvone was observed in the ASD group (*p* = 0.066).

For “mean duration of all explorations” (**Figures 4A–C**), there was a trend toward a lower value for m_Trimethylamine_ in ASD children than NT children, but analysis did not show any significant influence of group for m_g_, m_L-Carvone_, m_simple_, or m_mixture_. Moreover, intra-group comparison did not show any significant difference between Carvone and Trimethylamine, or between mixtures and their individual components, in the NT or ASD group.

Finally, for “duration of the first exploration” (**Figures 4D–F**), a significant effect of group was observed for m_Trimethylamine_, reflecting shorter exploration duration in ASD children than NT children, while no effect of group was observed for m_g_, m_L-Carvone_, m_simple_ or m_mixture_. Intra-group comparison revealed no significant differences between Carvone and Trimethylamine in the NT group, but a trend for ASD children to exhibit a shorter sniff in response to Trimethylamine than Carvone. No significant difference was observed between mixtures and their individual components, in the NT or ASD group.

**FIGURE 5:**
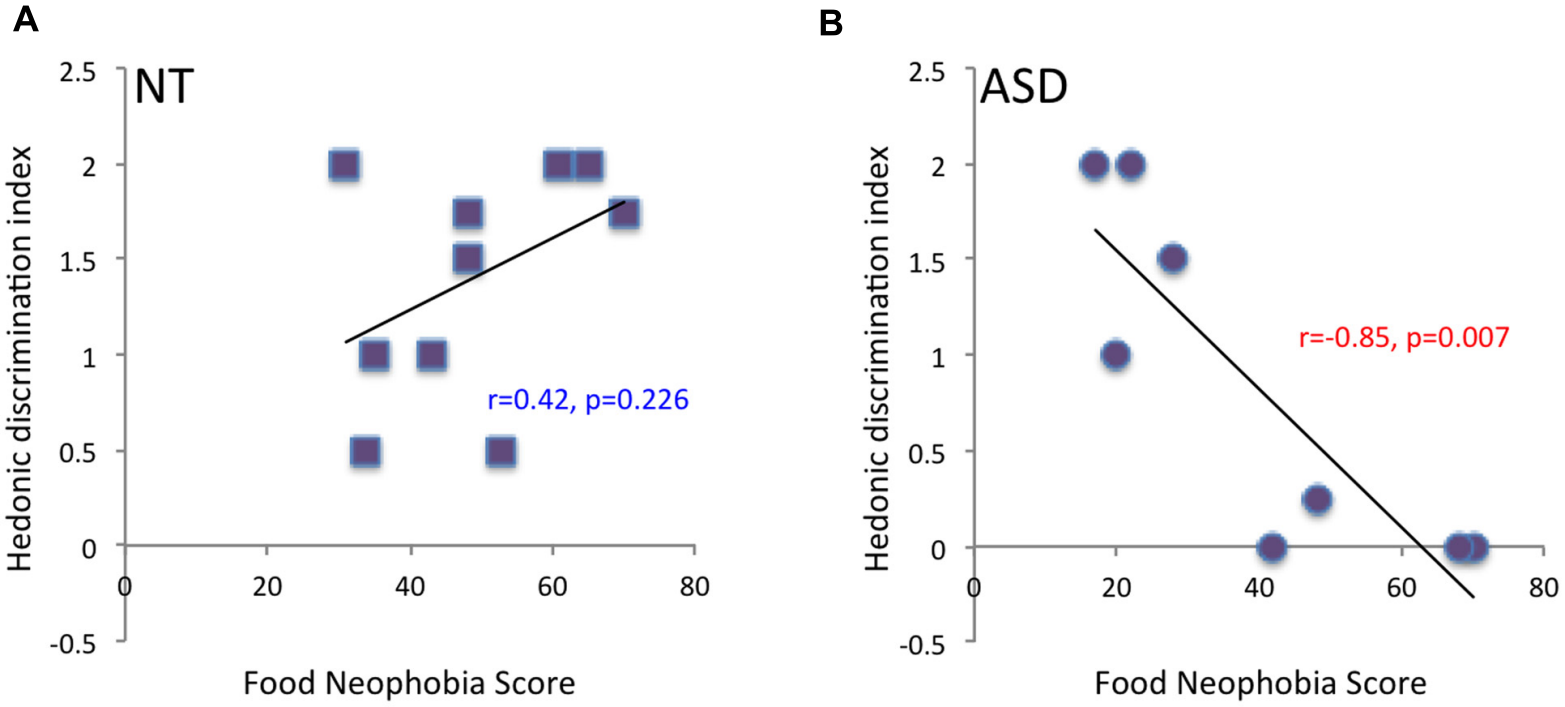
**Correlation between hedonic categorization index and food neophobia score.** A significant negative correlation between odor hedonic categorization index and food neophobia score was observed in ASD children (*p* = 0.006) **(B)**, but not in NT children **(A)**.

Thirdly, results regarding a link between odor pleasantness and food neophobia revealed no significant relationship between pleasantness ratings of unpleasant odors and food neophobia scores in NT (*r* = -0.27, *p* = 0.438) or ASD children (*r* = 0.33, *p* = 0.420). However, although there was no significant relationship between pleasantness ratings of pleasant odors and food neophobia scores in NT children (*r* = 0.28, *p* = 0.424), a trend toward a negative relationship was observed in ASD children (*r* = -0.65, *p* = 0.081): ASD children who perceived “attractive” odors as less pleasant had higher neophobia scores. This relationship between odor pleasantness and food neophobia in ASD children was confirmed by analysis taking account of the odor hedonic categorization index presented above: a significant negative relationship between odor hedonic categorization index and food neophobia score was observed in ASD (*r* = -0.85, *p* = 0.007) but not NT children (*r* = 0.42, *p* = 0.226): ASD children who had difficulty in hedonically categorizing smells (low index) had higher neophobia scores (**Figure 5**).

## Discussion

The aim of the present study was threefold: to examine whether ASD and NT children differed in odor perception, at both cognitive level (familiarity and identification ability) (objective 1) and sensorimotor (olfactory exploration) and hedonic levels (objective 2), and to assess a potential link between atypical odor perception and behavioral attitude toward food (food neophobia) (objective 3).

Regarding the **first objective**, the study provides very minor support for impaired odor identification in ASD children compared to controls: the only inter-group difference was that identification of the pleasant odor tended to be better than for the unpleasant odor in NT but not ASD children. Although studies have reported much evidence for impaired odor identification in ASD, findings have sometimes been inconsistent between studies. For example, Suzuki et al. (2003) measured odor detection and odor identification abilities in adult patients with Asperger’s syndrome and matched control subjects; compared to controls, patients exhibited intact odor detection levels but impaired odor identification ability. In another study, Galle et al. (2013) measured several aspects of olfactory perception (detection, discrimination, identification and ratings for intensity, pleasantness and familiarity) in ASD adults (including both classical autism and Asperger’s syndrome) and controls; whereas olfactory thresholds, odor discrimination and intensity, pleasantness and familiarity ratings did not differ between groups, odor identification ability was lower in autistic subjects than in both control and Asperger’s syndrome subjects.

Studies in ASD children reported inconsistent results. Bennetto et al. (2007), found that odor identification ability in ASD patients aged from 10 to 18 years old was lower than in controls. In a longitudinal study of ASD children, May et al. (2011) reported that odor identification ability improved with age (from 7 to 11 years) in ASD children as in controls. Dudova et al. (2011) reported that ASD children (mean age, 10 years) were impaired in odor detection as compared with matched controls, but not in identification ability (although ASD children identify the smell of orange better and the smell of cloves worse). Thus, identification ability does not seem to be clearly impaired in children with ASD, in line with the weak, non-significant difference in the identification performance between ASD and NT children in the present study. Nevertheless, it is worth mentioning here that it is not unlikely that both linguistic and cognitive factors characterizing the ASD group may have accounted for our findings. For example, language capacities were not measured and one cannot discard the possibility that odor identification performances in ASD children may depend on their level of language. Moreover, our group included Asperger’s syndrome participants whose performance could enhance the overall performance of the ASD group as suggested for adults by Galle et al. (2013).

With regard to the **second objective**, studies reported some minor differences in odor pleasantness in ASD children. For example, Hrdlicka et al. (2011), assessed differences in odor hedonic ratings in ASD children vs. controls. Odor hedonic ratings were measured on a 5-point scale using the smells contained in the identification part of the Sniffin Sticks test (see: Hummel et al., 1997; Kobal et al., 2000). The ASD children undervalued 2 of the 16 smells compared to controls, perceiving the odors of pineapple and cinnamon as less pleasant. It is worth noting that in a study with only ASD children, Dudova and Hrdlicka (2013) found no significant correlation between autism severity and odor detection, odor pleasantness ratings or odor identification ability. In the present study, whereas significant hedonic discrimination measured by verbal response (pleasantness of the attractive versus the aversive odor; **Figure 2B**) was observed in both groups, behavioral data (duration of first exploration) showed that ASD, unlike NT children, discriminated the unpleasant from the pleasant odor, the former being less explored (**Figure 4E**). This inconsistency between verbal reports and behavioral and implicit measures of olfactory processing was also noted by Legiša et al. (2013), who tested ASD children and matched controls (aged 8–14 years) and examined how emotional responses to odors were reflected in peripheral nervous system responses (facial and autonomic responses); the two groups showed very similar facial and autonomic emotional responses to smells but, comparing peripheral responses and verbal reports, ASD children seemed less likely to verbally express an affective state corresponding to their facial expression.

The **third objective** was to examine to what extent odor hedonics could be related to behavior toward food (i.e., food neophobia) in ASD children. Allowing for the limits related to the exploratory nature of the study, it emerged that less contrasted odor hedonic categorization was negatively correlated with food neophobia scores in ASD children: the less they discriminated hedonically (especially for pleasant odors), the more neophobic they were. Similarly, previous studies showed that difficulty in categorizing an object (e.g., food) was closely linked to its likability: the pleasantness or likability of foods that were difficult to categorize was diminished (Yamada et al., 2012). In the same study, food neophobia level was related to food likability. In agreement with such a link between odor hedonics and food neophobia, Raudenbush et al. (1998) showed that neophobic individuals evaluated smells as less pleasant and sniffed them less vigorously. In the present study, although food neophobia scores were similar in both groups, they were associated with different hedonic judgments between the two. It is known that children eat what they like and like what they know (Cooke et al., 2007). Therefore, given the significant influence of emotion on mnemonic processes (Kensinger, 2009a,b) and eating behavior (Aldridge et al., 2009), one hypothesis may be that the hesitation (or uncertainty) between a positive or negative judgment for emotional smells exhibited by certain ASD children influenced both acceptance of foods and neophobic construction. Although the present study does not provide significant proof of causality between differences in olfactory hedonics and food neophobia, our findings open up a new avenue of research in the field, considering the role of the olfactory function in understanding food neophobia construction in children with ASD. In addition, another future development regarding this issue could be the use of measurements that do not rely strongly on language and social capacities. Besides the behavioral characterization of children’s perception used here (number and duration of nasal explorations), it would be interesting to record physiological variables like sniffing, heart rate, respiratory rate, in order to strengthen our understanding of the relationship between food neophobia and affective perception of smells in ASD children.

While the present study provides new information about the olfactory function in ASD children, some of the methodological issues require discussion. For example, since most odorant molecules selected in the present study induce trigeminal sensations, one cannot discard the possibility that some differential effects between ASD children and controls are due to the stimulation of the fifth cranial nerve. Furthermore, it is important to note that this exploratory study comprised a small sample of subjects (10 per group). For practical reasons, it was not possible to include more participants in the study. Moreover, among ASD children, only 50% were able to complete the whole olfactory session. Differences between ASD children who could perform the entire study and those who could not, rely on cognitive, verbal and affective processing: (i) ASD children of our sample vary in their attentional abilities, some children being able to concentrate during the entire experimental task, and other not, (ii) one child who could not perform the entire study was non-verbal, (iii) some ASD children had strong emotional reactions following odor exposure, especially marked by disgust and aversion to some smells. These issues of exclusion of participants (two children for concentration and verbal problems), and missing data from 3 other children (particularly those who could not complete the entire task due to strong affective reactions to the smells) have an unknown impact on the study findings that extends beyond sample size and power limitations. Since not all children were able to test all 16 stimuli, additional analyses of the influence of odor intensity on odor pleasantness could not be assessed. Nevertheless, this issue provided important information about the number of stimuli that ASD children can experience in a reasonable amount of time (10 odorant conditions seems adequate according to the present findings). Another sample bias that may have affected some of the null findings is sample heterogeneity since our ASD group included six typical ASD children and four Asperger syndromes. It is likely that the use of a larger and less heterogeneous sample could have converted the few trends observed into significant effects. For example, the ability to identify an odor seems to be related to the degree of neophobia (Demattè et al., 2013), and this relationship deserves to be investigated further in larger groups of children. In particular, degree of neophobia is likely to be higher in ASD than NT children (Martins et al., 2008), which did not emerge in the present study likely because of lack of power.

In summary, notwithstanding the above, the present study offers new insights into odor perception in ASD children, highlighting a relationship between odor hedonic reactivity and eating behavior.

## Conflict of Interest Statement

The authors declare that the research was conducted in the absence of any commercial or financial relationships that could be construed as a potential conflict of interest.
